# Innovation and The Evolution of the Economic Web

**DOI:** 10.3390/e21090864

**Published:** 2019-09-05

**Authors:** Stuart Kauffman

**Affiliations:** Institute for Systems Biology, Seattle, WA 98109, USA; stukauffman@gmail.com

Fifty thousand years ago the global economy may have had a diversity of a few thousand goods and services, including fire, unifacial stone scrapers, hides, and so forth. Today, in New York alone, there must be over a billion goods and services. The global economy has exploded in diversity. The question is how and why has this explosion occurred?

The economy, as detailed a bit further below, is a network of complements and substitutes, which I will call the Economic Web. And like the biosphere, it’s evolution is substantially unprestatable, “context dependent,” and creates its own growing “context” that comprises its “Adjacent Possible.” The adjacent possible is what can arise next in this evolution. This evolution is “sucked into” the very opportunities it itself creates. Innovations into the Adjacent Possible drive this growth.

I do not wish to consider here the rich evolution of a single technology. Brian Arthur has brilliantly done so in his book *The Nature of Technology* [1]. Rather, I wish to discuss the evolution of the entire economic web, for as we shall see, goods and services create novel niches which invite the innovative creation of new complementary and substitute goods such that the web as a whole grows in diversity. 

## What is an Economic Web?

The two central ideas are complements and substitutes. A screw and a screwdriver are used together to create value, such as screwing in a screw. And so, they are complements. A screw and a nail can each be used to fasten two boards together. They are substitutes for each other. The economic web is the web of all goods and services, and for each, noted as a dot, a blue line connects it to all the other goods and services which are its complements and a red line connects it to all those other goods and services which are its substitutes. With billions of goods and services, this web is very complex indeed.

## Two Senses of “Need”

In addition to goods and services are needs. A first sense of “need” for a good can be a need for its complement. A screw “needs” a screwdriver to be of use in screwing in a screw. A second sense of need is that we humans often need to fasten things together. Ultimately, demand for a good or service depends upon our purposes and needs. The latter is the basis of utility theory in economics. Utility theory tries to define, often mathematically, the tradeoffs between goods from the point of view of a person, such the trade-off curve for consuming apples versus oranges given the preferences for each.

Economic opportunities typically exist for unmet needs in both senses of the word. Economists typically focus on the second sense, but sense 1 drives much of the evolution of the economic web, for a given technology *needs* its complements to be of use. So new technologies will drive economic growth by “needing” new complements and affording new opportunities to new substitutes. That need is an economic opportunity inviting innovations. In sense 2, we humans “need” word processing for its convenience in the preparation of documents. Thus, word processing emerged in the economic opportunity to fill that need, hence demand.

## A Brief Look at the Evolution of the IT Industry

The world of information technology has exploded in the past eighty years. In the 1930s, Turing invented the Turing Machine, an abstract formulation of a digital computer. By mid World War II, Turing’s idea was crafted at the University of Pennsylvania into the Eniac machine to calculate the trajectories of naval shells. After the war, von Neumann invented the framework for the mainframe computer and shortly later, IBM made the first commercial machines, expecting to sell only a few. But the main frame sold widely, and with the invention of the chip, its wide sale created a market that paved the way for the personal computer.

Note that the mainframe did not *cause* the invention of the personal computer, but the wide market the mainframe created *enabled* the rather easy penetration of the personal computer into an expanding market. In addition, the spreadsheet is often described in histories of technologies as the killer app that caused an explosion of the personal computer market. The spreadsheet is the complement of the personal computer. Each helped the other gain market share. 

The personal computer did not cause but enabled the innovation and invention of word processing, and software companies like Microsoft emerged, which was originally founded to make the operating system for IBM personal computers. 

The invention of word processing and abundant files invited the possibility of file sharing and the modem was innovated and invented. 

The existence of file sharing did not cause, but invited the innovation of the World Wide Web.

The existence of the Web did not cause, but enabled the innovation of selling on the Web and eBay and Amazon emerged.

eBay and Amazon put content on the Web as did myriad other users, enabling the innovations that were the invention of web browsers, and so companies like Google emerged. 

Thence, has followed social media and Facebook. 

Note, now that almost all of these successive innovations are the complement of the preceding ones. The existing goods and services at each state are the Actual “context” in which the next innovation of a new good and/or service emerges. Word processing is a complement of the personal computer, the modem a complement of word processing, the Web is a vast interconnected modem and is a complement and much more to file sharing. The opportunity to share files “invited” the innovative invention of the modem. 

I note again that goods and services as contexts do not cause but enable the innovation, invention and introduction of the next good or service. “Enablement” is not a word used in physics. The current Actual context of goods and services invites innovation into the current adjacent possible, of the next new goods and services, typically the complements and substitutes of existing goods and services.

A parallel history can be told of the automotive industry. The invention and advent of the automobile killed off the horse as a major mode of transport. With the horse, went the smithy, the buggy, the buggy whip, the corral. With the car came an oil and gas industry, paved roads, traffic control, motels, fast food restaurants, suburbia and people living in suburbia needing cars to get to work in town. Gas is the complement of the car, the motel is the complement of the car, and so forth. Each stage in that evolution begets the next stage. The Actual flows into its Adjacent Possible by the innovations for which the Adjacent Possible affords the new opportunities.

## The Adjacent Possible of the Economic Web

Given the main-frame and personal computer, word processing is an opportunity in the Adjacent Possible of the Economic Web. The Actual and the Adjacent Possible concern what exists now and what is next enabled to come to exist by the context of what is now Actual. What comes next in the Adjacent Possible emerges out of what is here now, i.e., the Actual. In general, the next evolution of the economic web grows out of whatever is now actual and flows into the adjacent possible that the current Actual enables. 

## The Algorithmic Adjacent Possible

Consider a Lego World. Start with a vast number of Lego blocks and place them on a central circle, ring 0, within a vast set of concentric circles like a target pattern. In ring 1, place all Lego objects that can be constructed from beginning Lego blocks in a single “legal” assembly, or “move,” like moves in a kind of chess game. In ring 2, place all objects constructable in two steps and so on to ring N out to infinity. The Lego block structures that exist “now” in, say, ring 7, unleash the Adjacent Possible of all the Lego structures that can next be constructed by single legal Lego moves.

This world is entirely “algorithmic” in the sense that there are “legal” Lego building moves. One may not, for example, use scotch tape to bind two blocks together instead of snapping them into place.

In a moment, we will see that the true Adjacent Possible of the economy is not algorithmic and not prestatable. 

New Goods, Services, and Production Functions Can Arise as New Combinations.

Consider the Wright brother’s airplane. It is a combination of a light gas engine, airfoil, bicycle wheels, and a propeller. The printing press was a recombination of a wine press and movable type. New goods are often such combinations. For example, a parachute on the back of a Cessna could become an airbrake. Arthur makes the same point in *The Nature of Technology* [1], Pietronero et al. mathematize this combinatorial accessing of the Adjacent Possible by innovations [2]. 

Therefore, new technologies grow out of the technologies that now exist. The Actual flows into its Adjacent Possible.

Thus, the economic web grows by creating its own “opportunities” to innovate and grow into the very Adjacent Possibilities it itself creates. 

More, the explosion of the goods and services in the global economy is largely due to the fact that the Adjacent Possible of the economy is itself growing rapidly. The current diversity of the economy abets the growing diversity of the next economy.

## The Non-Algorithmic Unprestatable Adjacent Possible of the Economy

LegoWorld is algorithmic with legal and non-legal moves of construction. The real economy is not so limited. Elsewhere, [3] I discuss the “screwdriver argument,” and jury rigging. I concluded that there is no algorithm that can list all the uses of a screwdriver, nor list the next use of a screwdriver. But we find new uses for screwdrivers all the time. I need merely recall James Bond in a crisis using a screwdriver to turn the situation to his advantage. 

But these new uses are typically unprestatable. 

More, these new uses are the very heart of innovation. 

This is now being recognized by industry. For example, consider “crowdsourcing”: Hey everyone, what is my new gadget useful for? 

Thus, the innovative new uses of things and processes, enabled by what is now actual, is how the economic web expands unprestatably into its Adjacent Possible.

A charming true story exemplifies all of this. A few years ago, a man was living in Tokyo around the time the iPhone was introduced. He lived in a tiny apartment with a new baby, and it was crowded by his many books. He realized he could copy all his books with his iPhone then sell the books to create more space in his apartment. Then, he realized his opportunity. Many other families in Tokyo lived in crowded apartments, many with books. He could go to these and offer to use his iPhone to copy their books, sell them and take a percentage of the sale as his profit! His business was a success and is now itself being copied. What was his opportunity? Crowded apartments, the iPhone, and markets for books. The new business was his innovation.

We come to an important conclusion: The growth of the economic web is “sucked into” the very Adjacent Possible it itself creates by the innovations it invites!

## The Unknowable “Size” of the Adjacent Possible

We cannot measure the “size” of the adjacent possible. We do not know what is in it. Consider flipping a fair coin 1000 times and asking if it will come up heads 540 times. We do not know, but can calculate the probability with the binomial theorem. We do not know what *will* happen, but we know what *can* happen. We know all two to the 1000 possible outcomes of flipping a fair coin 1000 times. We know the sample space of the process.

But for the evolution of the economy into its Adjacent Possible, we do not know the sample space! Hence, we can construct no probability measure. Therefore, we cannot know the size of the Adjacent Possible.

## The Diversity of Contexts and Diversity of Uses

The number of uses of a screwdriver depends upon the diversity of the context. A screwdriver cannot be used to do much by itself in empty space, but can be used to do many things alone or with other things in New York in 2019.

Consider jury rigging. There can be no deductive theory of jury rigging. Hence, there can be no deductive theory of innovation! But it seems we can say something. If you confront some arbitrary problem, would you be better off jury rigging with a single object or process, say a screwdriver, or an assembly of many objects, screwdriver, duct tape, a shoe horn, old battery, bailing wire, nails, a sheet of cloth.

Obviously, it is easier to jury rig with lots of objects lying to hand than a single object. While we seem unable to quantify this, at least now, it seems clearly true. 

In short, the diversity of “context”—here, the number of objects available—is related to the number of “things” one can do with the assembly. A garage full of stuff is more easily commandeered to new ends that a clean one. 

## The Growing Web *is* the Growing Context For its Own Further Growth

As new goods and services and production capacities come into existence, they provide the growing contexts into which yet more new goods and services and production capacities can follow as their complements or substitutes. An economy with a high diversity of goods, services and production functions is rather like a garage full of “stuff,” rather than a clean garage. It is easier to jury rig and innovate in the garage full of stuff and it is easier to invent new goods and services and production functions in an economy already full of such stuff. But the new goods, services and production functions only make the “garage” more full of stuff, thus, amazingly, the economy grows its own adjacent possible and *augments* that very growth as the growth occurs. The process is broadly self-accelerating. 

Thus, the growing economic web explodes in diversity of complements and substitutes from perhaps a thousand or ten thousand goods 50,000 years ago to billions today!

But the same holds true for the evolving biosphere. This is seen in the expanding diversity of species in the Phanerozoic in the last 600 million years. New species literally create niches for yet further new species. New goods create niches for yet further new goods and services and production capacities. 

## A Brief Comment on Standard Economic Growth Models

What I have sketched here is very different from most standard economic growth models. These model the economy, not as a web, but a single sector making, in effect, a single product. Then, one considers input factors such as capital and labor and human knowledge, investment, and saving and writes differential equations which can model growth. These work fine to some extent, but not for an economy which is creating ever new goods and services as is our economic web. 

## An Early Statistical Model of the Adjacent Possible

We have, at present, no mathematical model for the unprestatable evolution I describe above. However, S. Strogatz and V. Loreto [4] have taken an important first step. Theirs is a first model of the Adjacent Possible. They start with what is known in mathematics as a Polya urn model. In this model, one starts with an urn holding 50 percent black balls and 50 percent white balls. The player randomly picks a ball. If it is white (or black), he replaces the ball and adds one white (or black) ball. The question is, after a long time, what is the stationary fraction of white balls. The answer is, “any value between 0 percent and 100 percent equi-probably. That is, one could have 69 percent black balls and 31 percent white balls, or 0 percent black balls and 100 percent white balls. 

In Strogatz and Letto’s variation, one starts with at least two colors of balls. All picked balls are replaced in the urn. But if a color is picked that was not seen before, it is replaced, and a random *new* colored ball is introduced. The new color models innovation into the new adjacent possible. The process continues indefinitely. The process generates a power law distribution of colors and fits both Zipf’s law and Heap’s law. The random new colors are an initial move to model the unknowable adjacent possible. Their fit to both Zipf’s law and Heap’s law with much data is encouraging.

The model is lovely, but does not yet answer our needs, for it is one of a branching set of independent lineages of descendant colored balls. A red ball gives rise to an orange ball gives rise to a blue ball. There is no cross talk between lineages augmenting the combinatorial formation of new colors as there is in the economic web’s evolution with new complements and substitutes arising from old ones by new jury-rigged combinations of one or several prior goods. I hope that a good model or set of models can be constructed. 

The evolution of the economy parallels the unentailed evolution of the biosphere as species create niches for one another, often adaption of the evolving biosphere. In both cases, like the garage filling up with ever newly invented “stuff” for jury-rigging, life creates its own staggering possibilities of future becoming. 

To think that this evolution is a Newtonian–Laplacian machine, derivable in it specific becoming from some set of axioms, seems deeply wrong. Life, and we and our evolving economy among it, is so rich in its inheritance and prospects that we can, I think, be captured by no entailing laws. 
